# Hepatic Polarization Accelerated by Mechanical Compaction Involves HNF4*α* Activation

**DOI:** 10.1155/2020/8016306

**Published:** 2020-08-05

**Authors:** Jinlian Yang, Jiaen Liang, Yongjian Zheng, Shiying Li, Yang Li, Haiyan Liu, Guanzhong Chen, Jing Ma, Ziyu Liao, Jiezhao Lin, Zesheng Jiang, Yan Wang

**Affiliations:** ^1^State Key Laboratory of Organ Failure Research, Guangdong Provincial Research Center for Liver Fibrosis, Department of Infectious Diseases and Hepatology Unit, Nanfang Hospital, Southern Medical University, Guangzhou, Guangdong 510515, China; ^2^Biomedical Research Center, Southern Medical University, Guangzhou, Guangdong 510515, China; ^3^School of Pharmaceutical Sciences, Southern Medical University, Guangzhou, Guangdong 510515, China; ^4^Department of Hepatobiliary Surgery, Zhujiang Hospital, Southern Medical University, Guangzhou, Guangdong 510280, China

## Abstract

There remain few data about the role of homeostatic compaction in hepatic polarization. A previous study has found that mechanical compaction can accelerate hepatocyte polarization; however, the cellular mechanism underlying the effect is mostly unclear. Hepatocyte nuclear factor 4 alpha (HNF4*α*) is crucial for hepatic polarization in liver morphogenesis. Therefore, we sought to identify any possible involvement of HNF4*α* in the process of hepatocyte polarization accelerated by mechanical compaction. We first verified in the nonhepatic cell model HEK-293T, and the hepatic cell model primary hepatocytes that the mechanical compaction on cell aggregates simulated by using transient centrifugation can directly activate the expression of HNF4*α* promoters. Moreover, data using primary hepatocytes showed that the HNF4*α* expression is positively associated with the levels of compaction force: 2.1-folds higher at the mRNA level and 2.1-folds higher at the protein level for 500 g vs. 0 g. Furthermore, activated HNF4*α* expression is associated with the enhanced biliary canalicular formation and the increased production of albumin and urea. Pretreatment with Latrunculin B, an inhibitor of F-actin, and SHE78-7, an inhibitor of E-cadherin, which both interrupt the pathway of mechanical transduction, partially but significantly reduced the HNF4*α* expression and production of albumin and urea. In conclusion, HNF4*α* can be actively involved in the hepatic polarization in the context of environmental mechanical compaction.

## 1. Introduction

Hepatic polarity is important for the generation and maintenance of physiological function in hepatocytes [1]. It also contributes to diverse pathological processes, such as hepatitis B virus cellular intrusion, intrahepatic cholestasis, and steatosis [2, 3]. However, the mechanism underlying the maintenance of polarity in hepatocytes in mature liver tissues is far from clear. A previous study has found that mechanical compaction accelerated hepatocyte repolarization and bile canaliculus (BC) formation by imposing extensive intercellular contact and adhesion [4]. Mechanical compaction accelerated hepatocyte repolarization without impairing the hepatocytes' remodeling and functional capabilities. However, cellular mechanism underlying this interesting effect is yet to be understood.

Recently, it became evident that mechanical forces applied by the pericellular environment or generated inside cells function as important upstream signals that trigger the establishment of cell and tissue polarity [5–7]. For instance, for the Drosophila wing, disc planar cell polarity proteins coordinately reorient in line with stress patterns induced by hinge contraction [8], and for endothelial cells, shear stress regulates the forward and the reverse planar cell polarity of the vascular endothelium through upregulation of glycogen synthase kinase-3 in vivo and in vitro [9]. To respond to various mechanical forces, cells may sense and translate mechanical forces into a biochemical signal by cellular elements such as the actin cytoskeleton and cadherin-mediated cell adherent junctions [10].

Hepatocyte nuclear factor 4 alpha (HNF4*α*), an orphan member of the nuclear receptor superfamily (NR2A1), is physiologically expressed at a high level and binds to the promoters of 12% of genes in the adult liver [11]. A role for HNF4*α* in hepatic polarity was suggested because its deficiency in the embryonic mouse liver resulted in an abnormal tissue architecture and a lack of cell polarity [12]. Its overexpression induces cell polarity in F9 embryonic carcinoma [13] and H5 hepatoma [14]. Although the effects of the mechanical force on the establishment and maintenance of cell polarization have been generally recognized [5–7], whether there could be an involvement of HNF4*α* in the accelerated hepatic polarization by mechanical compaction still lacks supporting evidence. Therefore, in the present study, we sought to investigate (1) whether mechanical compaction can directly enhance HNF4*α* expression and (2) whether the cytoskeleton as well as cadherin adherent junctions is involved in the process.

## 2. Materials and Methods

### 2.1. Primary Hepatocyte Isolation and Maintenance

Adult male wild-type C57BL/6 mice (6-8 weeks old) were purchased from the Laboratory Animal Center of Guangdong Province. Primary hepatocytes were isolated from C57BL/6 mice via collagenase perfusion as described previously [15]. Cells were cultured in William's E medium (Sigma-Aldrich, St. Louis, MO) supplemented with 1 mg/mL bovine serum albumin (Mp Biomedicals, California, USA), 5 ng/mL epidermal growth factor (Prospec, Saint Louis, USA), 0.2 units/mL insulin (Sigma-Aldrich), 100 nmol/L dexamethasone (Sigma-Aldrich), and penicillin/streptomycin (Sigma-Aldrich). Prior to seeding, the cell culture substrates were coated with 0.012 mg/mL acidic collagen (1 : 417 dilution of PureCol® collagen (Advanced BioMatrix, USA) in 6 mM acetic acid). The cells were then seeded onto the collagen-coated membrane and maintained in a 5% CO_2_ atmosphere at 37°C. Culture medium was changed every day. All animal experiments were previewed and approved by the Animal Care and Use Committee of Southern Medical University.

### 2.2. Cell Culture

Human cell lines HepG2 (ATCC, HB-8065) and HEK-293T (ATCC, CRL-11268) were purchased from the American Type Culture Collection (Manassas, VA, USA). They were grown in DMEM (HyClone, Logan, UT, USA) supplemented with 4.5 g/L glucose, 10% fetal bovine serum (HyClone), 100 U/mL penicillin, and 100 *μ*g/mL streptomycin. Cells were maintained in 5% CO_2_ at 37°C. Culture medium was refreshed every 2 to 3 days.

### 2.3. Luciferase Reporter Assay

Cells cultured in 6-well plates were transfected with Gaussia Luciferase (GLuc) and Secreted Alkaline Phosphatase (SEAP) promoter reporter clones containing the HNF4*α* promoter 1 (P1, Cat. no. HPRM16317-PG04) or P2 (Cat. no. HPRM20338-PG04) gene sequences (GeneCopoeia, Germantown, MD) by using Lipofectamine 2000 (Invitrogen, Carlsbad, CA, USA). Culture medium was collected at 24 h after transfection. GLuc and SEAP activities were measured using the Secrete-Pair Dual Luciferase Assay Kit (GeneCopoeia) according to the manufacturer's instructions. Data were normalized against SEAP. A GLuc and SEAP promoter reporter clone with the glyceraldehyde 3-phosphate dehydrogenase (GAPDH) gene sequence insertion was used as a positive control, and a wild-type GLuc and SEAP promoter reporter clone was used as the negative control group (NEG).

### 2.4. Mechanical Compaction on the Cell Seeding

**Figure 1:**
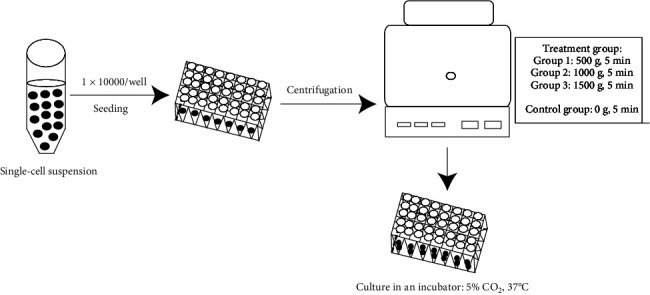
The schematic diagram of mechanical compaction on cells.

We used instant centrifugation to simulate the mechanical compaction on cell seeding, as centrifugation can generate the compaction force between the cells, and more importantly, it can quantify the force. The schematic diagram of mechanical compaction on cells is shown in Figure 1. In brief, single-cell suspension was seeded into V-bottom 96-well plates and centrifuged for 5 min at 0 × *g*, 500 × *g*, 1000 × *g*, and 1500 × *g*. Cells were maintained in 5% CO_2_ at 37°C and then were cultured. The culture medium was collected, and the GLuc and SEAP activities of HNF4*α* P1 and P2 were assessed at 0 h, 3 h, 6 h, 12 h, and 24 h after centrifugation. The total RNA and total protein in cells were extracted to measure the mRNA and the protein expression levels of HNF4*α*. Cells centrifuged at 0 × *g* were used as the control. Since 500 × *g* had the most significant effect, it was used in subsequent experiments.

### 2.5. Disruption of the Polymerization of Cellular F-Actin

Cells were cultured with 1000 and 2000 nM Latrunculin B (LatB; Cat. no. ab144291, Abcam, Cambridge, England) for 2 h to disrupt the polymerization of cellular F-actin [16]. The morphology of F-actin polymerization was observed after specifically stained F-actin with Alexa Fluor 488 phalloidin (Cat. no. A12379, 1 : 20, Invitrogen) by using a Leica DM2500 fluorescence microscope with a 20x objective. Since 2000 nM LatB had the better effects, it was used in the mechanical compaction and its subsequent experiments.

### 2.6. Disruption of the Establishment of Cadherin Adherent Junction

Cells were treated with 7.5 and 15 *μ*g/mL SHE78-7 (Cat. no. 135700, Invitrogen) for 24 h to disrupt the establishment of cadherin adherent junction [17]. The E-cadherin adherent cell-cell contacts were observed by using an inverted-phase contrast microscope with a 20x objective. Since 15 *μ*g/mL SHE78-7 had the better effects, it was used in the mechanical compaction and its subsequent experiments.

### 2.7. Albumin and Urea Secretion Assays

Culture media were collected at 0 h, 3 h, 6 h, 12 h, and 24 h from each well and stored at -20°C until the hepatic function assay was performed. The albumin and the urea production were quantified using a fully automatic biochemistry analyzer (Olympus Au400; Tokyo, Japan).

### 2.8. Transmission Electron Microscopy (TEM)

Cells were fixed with 2.5% glutaraldehyde containing 0.1 mol/L sodium cacodylate and treated with 1% osmium tetroxide. After dehydration, samples were embedded in araldite, then cut into thin sections and stained with uranyl acetate and lead citrate. Digital images were obtained using a Hitachi H-7500 transmission electron microscope operated at 80 kV.

### 2.9. Western Blot Analysis

The expression level of HNF4*α* protein was measured by western blotting as previously described [18]. The following antibodies were used: primary antibodies HNF4*α* (Cat. no. ab201460, 1 : 1000, Abcam) and GAPDH (Cat. no. KC-5G5, 1 : 8000, KangChen Bio-tech, Shanghai, China) and an HRP-conjugated secondary antibody (Cat. no. bs-0295G-HRP, 1 : 8000, Boster, Wuhan, China). Endogenous GAPDH was used as a loading control. The X-ray film of the internal reference control GAPDH and the target protein HNF4*α* band was photographed using a gel image scanning analysis system. The integrated optical density value of each band was measured by using the gray-scale analysis software Image-Pro Plus 6.0. The optical density ratio of the target protein/internal reference protein is the relative expression of the target protein.

### 2.10. RNA Extraction and Real-Time Polymerase Chain Reaction (RT-PCR) Analysis

Total RNAs from cell cultures were lysed using TRIzol® Reagent (Takara, Dalian, China), and genomic DNA was removed using the gDNA Eraser (Takara). RNA was reversely transcribed using RT Primer Mix and PrimeScript RT Enzyme Mix I (Takara). Expression analysis was performed by SYBR Green-based RT-PCR using SYBR® Premix Ex Taq II (Tli RNaseH Plus, Takara) and the 7500 RT-PCR Systems (Foster City, CA). Primers used for genes of interest were purchased from GeneCopoeia. Results were calculated using Ct values and normalized against endogenous *GAPDH* mRNA levels.

### 2.11. Generation of Transient Cell Lines with Enhanced HNF4*α*

Nowadays, there is no relevant literature report on which the HNF4*α* isoform regulates the polar phenotype of liver epithelial cells. We searched for transcripts of HNF4*α* in the National Center for Biotechnology Information gene library and found that the transcript NM-000457.5 which encodes HNF4*α*2 is the longest in the all isoform transcripts, which means the transcript NM-000457.5 carries and expresses the most information of HNF4*α* compared with other isoform transcripts. Moreover, the transcript NM-000457.5 covers 71-86% of the complete sequence of the different HNF4*α* isoforms and contains the C, D, and E domains of HNF4*α*. In addition, in terms of the transcriptional function of the HNF4*α* isoforms, the *α*1 and *α*2 isoforms were the strongest regulators of gene expression [19]. Therefore, we choose to overexpress and silence the HNF4*α*2 isoform in the HepG2 cells to further explore the function of HNF4*α* in the polar phenotype of liver epithelial cells.

To establish cell lines with enhanced HNF4*α*, the recombinant adenovirus containing HNF4*α*2 gene sequences (Ad-HNF4*α*; Cat. no. HH20150327LJM-Ap01, Hanbio Biotechnology, Shanghai, China) or a negative control adenovirus (Ad-Mock, Hanbio Biotechnology) was used to infect HepG2 cells. The HNF4*α* expression was determined at the mRNA and protein levels.

### 2.12. Generation of Stable Cell Lines with Suppressed HNF4*α*

To establish cell lines with suppressed HNF4*α*, the recombinant lentivirus containing oligonucleotides encoding a short hairpin RNA targeting HNF4*α*2 (LV-HNF4*α*; Cat. no. HH20150316LJM-Lv01, Hanbio Biotechnology) or a negative control lentivirus (LV-Mock, Hanbio Biotechnology) was used to infect HepG2 cells with polybrene (Cat. no. H9268, Sigma-Aldrich). After puromycin (Cat. no. A11138, Invitrogen) selection for 2 weeks, the stable clones were obtained. The HNF4*α* expression was determined at the mRNA and protein levels.

### 2.13. Functional Analysis of HepG2 Polarity

Briefly, cells were washed with fresh medium, incubated with medium containing 20 *μ*g/mL fluorescein diacetate (Cat. no. F7378, FDA, Sigma-Aldrich) for 40 min at 37°C, and viewed using a Carl Zeiss LSM880 confocal microscope with a 60x oil objective within 5 min. The mean areas of FDA localization were identified and quantified five nonoverlapping fields of view using Image-Pro Plus 6.0 software.

### 2.14. Statistical Analysis

All data are expressed as means ± standard deviation (means ± S.D.) of three independent experiments. GraphPad Prism 5.01 and Adobe Illustrator CS6 were used to generate plots and perform statistical analyses. Data were analyzed using either one-way analysis of variance (Dunnett's multiple comparison test) or two-way analysis of variance (Bonferroni posttests). A level of *P* < 0.05 was considered statistically significant.

## 3. Results

### 3.1. Mechanical Compaction Activates HNF4*α* Expression

**Figure 2:**
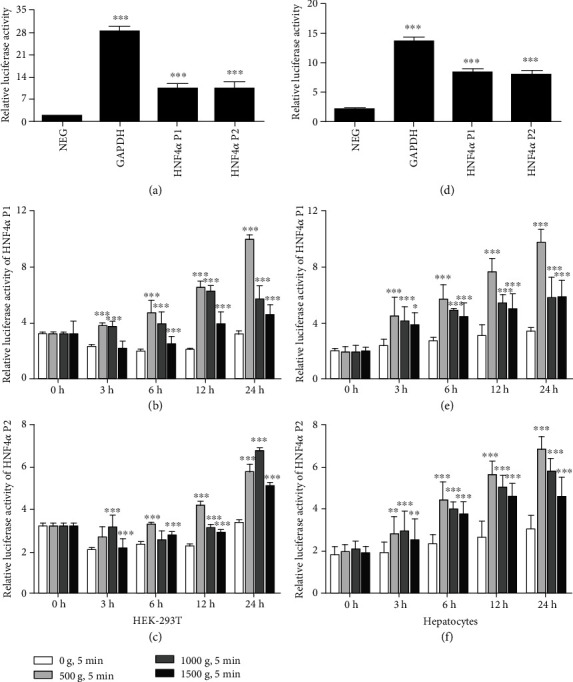
Mechanical compaction activates HNF4*α* P1 or P2 expression in HEK-293T cells or primary hepatocytes. (a, d) Relative luciferase activities of cells transfected with clones containing HNF4*α* P1 or P2, GAPDH, and NEG (one-way ANOVA analysis and Dunnett's multiple comparison test, HNF4*α* P1 and P2, GAPDH vs. NEG, *n* = 3, ^∗∗∗^*P* < 0.001). (b, c, e, f) The relative luciferase activities of HNF4*α* P1 and P2 in HEK-293T cells or primary hepatocytes treated by centrifugation at 500-1500 × *g* (two-way ANOVA analysis and Bonferroni posttests, 500-1500 g vs. 0 g, *n* = 3, ^∗^*P* < 0.05, ^∗∗^*P* < 0.01, ^∗∗∗^*P* < 0.001).

The initiation of HNF4*α* gene expression in liver cells mainly occurs by the activation of HNF4*α* P1 or P2 [20]. The renal epithelial HEK-293T cells were first treated to avoid the effect of endogenous HNF4*α*. As shown in Figure 2(a), the relative luciferase activity of HEK-293T cells transfected with GLuc and SEAP promoter reporter clones containing the HNF4*α* P1 gene sequences was 5.9-folds higher (*P* < 0.001) compared with that of the NEG. The relative luciferase activity of cells transfected with clones containing the HNF4*α* P2 gene sequences was 6.2-folds higher (*P* < 0.001). In the positive control cells transfected with the clones containing GAPDH gene sequences, the relative luciferase activity was 14.8-folds higher (*P* < 0.001).

Then, single HEK-293T cell suspension transfected with GLuc and SEAP promoter reporter clones containing the HNF4*α* P1 or P2 gene sequences was seeded into V-bottom 96-well plates, simulated by using transient centrifugation before static culture. The viabilities of HEK-293T cells after centrifugation did not change significantly during the 24 h (Figure S1). As shown in Figures 2(b) and 2(c), compared with the control group (0 × *g*), the relative luciferase activities of HNF4*α* P1 and P2 in cells centrifugated by 500 × *g* were, respectively, 1.6- (*P* < 0.001) and 1.3- (*P* = 0.065) folds higher at 3 h and increased to 3.1- (*P* < 0.001) and 1.9- (*P* < 0.001) folds higher at 24 h. In cells centrifugated by 1000 × *g*, the relative luciferase activities of HNF4*α* P1 and P2 were, respectively, 1.6- (*P* < 0.001) and 1.5- (*P* < 0.001) folds higher at 3 h and increased to 3.0- (*P* < 0.001) and 2.0- (*P* < 0.001) folds higher at 24 h. In cells centrifugated by 1500 × *g*, the relative luciferase activities of HNF4*α* P1 and P2 were equal to those in the control cells at 3 h and increased to 1.4- (*P* < 0.001) and 1.5- (*P* < 0.001) folds higher at 24 h, respectively. The relative luciferase activities of HNF4*α* P1 or P2 increased gradually during the culture period.

We applied the same treatment to primary hepatocytes, and the relative luciferase activities of HNF4*α* P1 and P2 were similar to those in the HEK-293T cells. Compared with the control group (0 × *g*), the HNF4*α* P1 or P2 relative luciferase activities in primary hepatocytes stimulated by 500 × *g*, 1000 × *g*, or 1500 × *g* were 1.3–1.9-folds higher at 3 h (*P* < 0.05) and increased to 1.5–2.9-folds higher at 24 h (*P* < 0.001, Figures 2(d)–2(f)).

**Figure 3:**
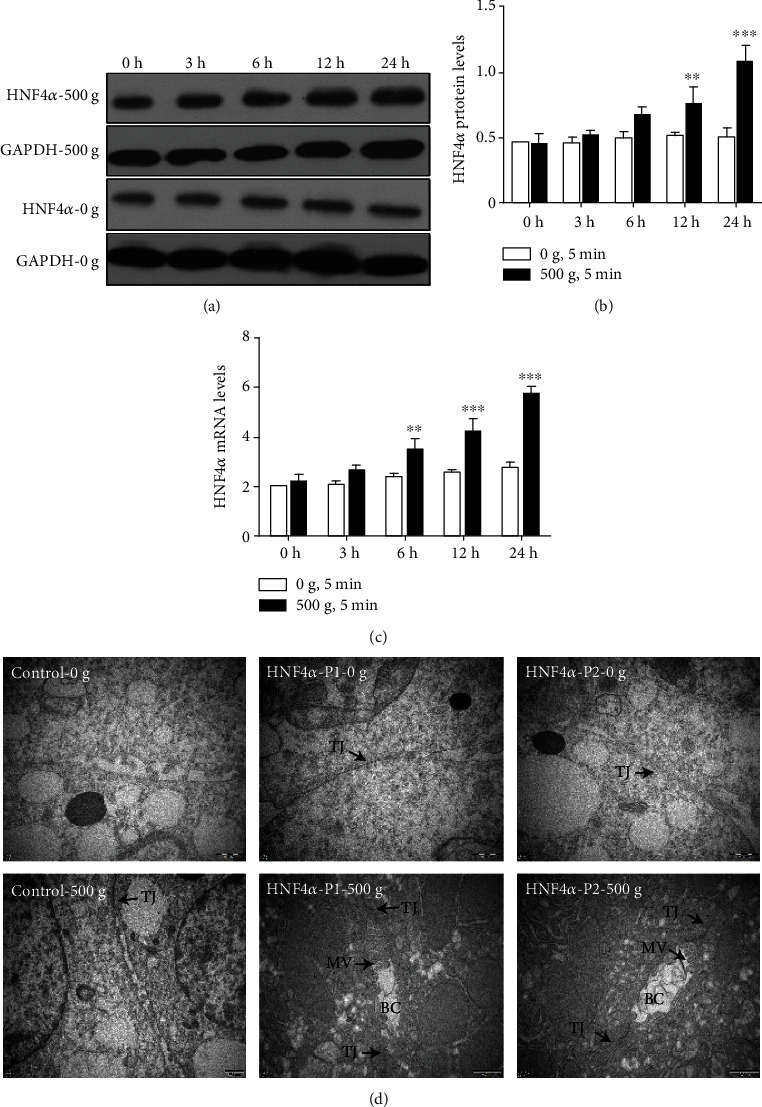
Mechanical compaction enhances HNF4*α* expression and accelerates BC formation in primary hepatocytes. (a–c) The mRNA and protein levels of HNF4*α* increased over time in the primary hepatocytes stimulated by 500 × *g* (two-way ANOVA analysis and Bonferroni posttests, 500 g vs. 0 g, *n* = 3, ^∗∗^*P* < 0.01, ^∗∗∗^*P* < 0.001). (d) Ultrastructural analysis of primary hepatocytes treated with 0 × *g* or 500 × *g* and then cultured for 24 h. Bile canaliculi (BC), tight junctions (TJ), and microvilli (MV) are indicated by arrows. Scale bars: 500nm.

Moreover, compared with the control group (0 × *g*), the HNF4*α* mRNA and protein expression levels in primary hepatocytes stimulated by centrifugation at 500 × *g* were equal to those in the control cells at 3 h and increased to 2.1- (*P* < 0.001) and 2.1- (*P* < 0.001) folds higher at 24 h (Figures 3(a)–3(c)), respectively.

Collectively, mechanical compaction can effectively activate HNF4*α* expression. Since 500 × *g* had the most significant effect, it was used in the subsequent experiments.

### 3.2. Mechanical Compaction Accelerates BC Formation and Enhances Primary Hepatocyte Functions

Here, we used BC formation to exemplify the formative process of functional structures in the primary hepatocyte. Primary hepatocytes were transfected with clones containing the HNF4*α* P1 or P2 gene sequences, seeded into V-bottom 96-well plates, simulated by 500 × *g*, and placed on static culture for 24 h. Then, the ultrastructures of the primary hepatocytes were observed by TEM. As shown in Figure 3(d), we found that the BC cavities, microvilli, and tight junctions were present in the primary hepatocytes stimulated by 500 × *g* and absent in the control group cells stimulated by 0 × *g*.

**Figure 4:**
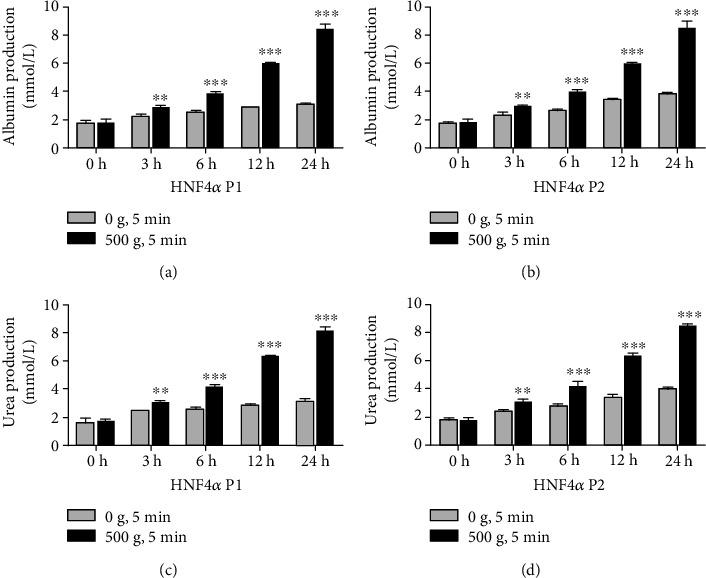
Mechanical compaction enhances primary hepatocyte functions. (a–d) The expression levels of albumin and urea production in the primary hepatocytes stimulated by 500 × *g* were significantly elevated and were higher than those in the control cells (two-way ANOVA analysis and Bonferroni posttests, 500 g vs. 0 g, *n* = 3, ^∗^*P* < 0.05, ^∗∗^*P* < 0.01, ^∗∗∗^*P* < 0.001).

The albumin and the urea production are important indicators of hepatocyte functions during in vitro culture [21, 22]. Compared with the control group (0 × *g*), albumin production in the primary hepatocytes transfected with clones containing the HNF4*α* P1 or P2 gene sequences and stimulated by 500 × *g* was, respectively, 1.3- (*P* < 0.01) and 1.3- (*P* < 0.01) folds higher at 3 h and increased to 2.8- (*P* < 0.001) and 2.2- (*P* < 0.001) folds higher at 24 h (Figures 4(a) and 4(b)). And urea production was, respectively, 1.2- (*P* < 0.01) and 1.3- (*P* < 0.01) folds higher at 3 h and increased to 2.6- (*P* < 0.001) and 2.1- (*P* < 0.001) folds higher at 24 h (Figures 4(c) and 4(d)). Both the albumin and the urea production increased gradually during the culture period.

### 3.3. Disruption of F-Actin Polymerization Partially Suppresses HNF4*α* Expression Stimulated by Mechanical Compaction

**Figure 5:**
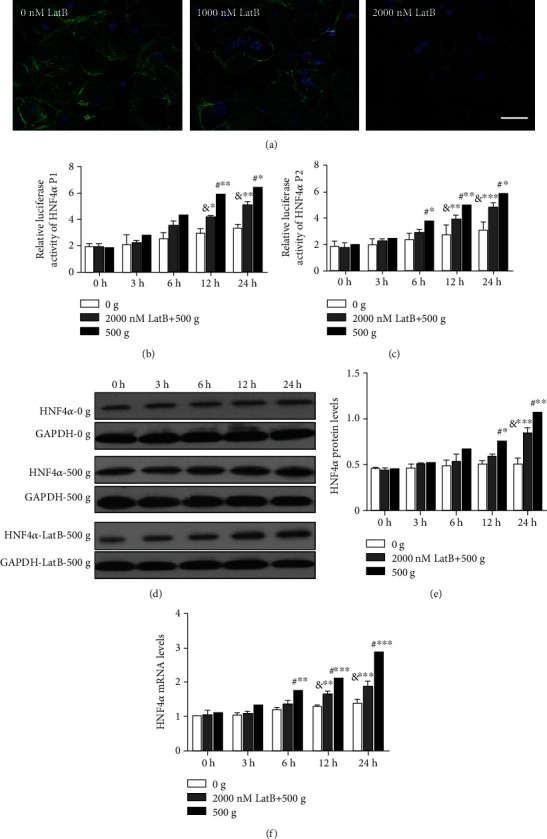
Disruption of F-actin polymerization partially but significantly reduced the relative luciferase activities of HNF4*α* P1 and P2 and the expression levels of HNF4*α* induced by mechanical compaction. (a) LatB effectively disrupted F-actin polymerization in subconfluent cell cultures. (b, c) The relative luciferase activities of HNF4*α* P1 and P2 and (d–f) the mRNA and protein levels of HNF4*α* in primary hepatocytes exposed to LatB and stimulated by 500 × *g* were lower than those in cells stimulated by 500 × *g* and untreated with LatB but still higher than those in cells just stimulated by 0 × *g* (two-way ANOVA analysis and Bonferroni posttests, ^&^LatB treated vs. 0 g, ^#^LatB treated vs. 500 g, *n* = 3, ^∗^*P* < 0.05, ^∗∗^*P* < 0.01, ^∗∗∗^*P* < 0.001).

In the control primary hepatocytes, the distribution of F-actin showed a typical network of microfilament bundles (stress fibers). In the primary hepatocytes treated with 1000 nM LatB, the contraction of F-actin microfilament bundles was not complete and some stress fibers disappeared. In the primary hepatocytes treated with 2000 nM LatB, contraction of F-actin microfilament bundles was complete and most of the stress fibers disappeared (Figure 5(a)). Consistent with previous results [23], LatB at 2000 nM had a better suppressive effect on F-actin polymerization and was used in the subsequent experiments.

In the primary hepatocytes treated with 2000 nM LatB and stimulated by 500 × *g*, the relative luciferase activities of HNF4*α* P1 were 19.2% (*P* = 0.089) lower than those in the cells stimulated by 500 × *g* and untreated with LatB at 3 h and 20.9% (*P* < 0.05) lower at 24 h (Figure 5(b)). Similarly, the relative luciferase activities of HNF4*α* P2 were 11.8% (*P* = 0.093) lower at 3 h and 18.5% (*P* < 0.05) lower at 24 h (Figure 5(c)).

Moreover, compared with the control cells stimulated by 500 × *g* and untreated with LatB, the protein levels of HNF4*α* in primary hepatocytes exposed to 2000 nM LatB and stimulated by 500 × *g* were 2.0% (*P* = 0.096) lower at 3 h and 21.7% lower (*P* < 0.01) at 24 h (Figures 5(d) and 5(e)). The mRNA levels of HNF4*α* were 17.7% (*P* = 0.059) lower at 3 h and 34.8% (*P* < 0.001) lower at 24 h (Figure 5(f)).

### 3.4. Disruption of E-Cadherin-Mediated Cell Adherent Junctions Partially Suppresses HNF4*α* Expression Stimulated by Mechanical Compaction

**Figure 6:**
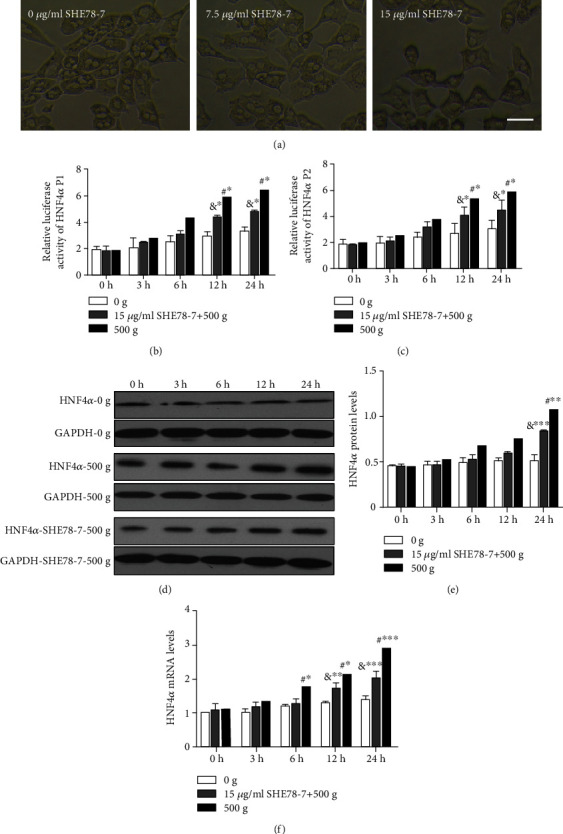
Disruption of E-cadherin-mediated cell adherent junctions partially but significantly reduced the relative luciferase activities of HNF4*α* P1 and P2 and the expression levels of HNF4*α* induced by mechanical compaction. (a) SHE78-7 effectively disrupted cell-cell contacts in subconfluent cell cultures. (b, c) The relative luciferase activities of HNF4*α* P1 and P2 and (d, f) the mRNA and protein levels of HNF4*α* in primary hepatocytes exposed to SHE78-7 and stimulated by 500 × *g* were lower than those in cells stimulated by 500 × *g* and untreated with SHE78-7 but still higher than those in cells just stimulated by 0 × *g* (two-way ANOVA analysis and Bonferroni posttests, ^&^SHE78-7 treated vs. 0 g, ^#^SHE78-7 treated vs. 500 g, *n* = 3, ^∗^*P* < 0.05, ^∗∗^*P* < 0.01, ^∗∗∗^*P* < 0.001).

In the control primary hepatocytes, the cadherin adherent junction showed a typical morphology. In the primary hepatocytes treated with 7.5 *μ*g/mL SHE78-7, the cadherin adherent junctions were lost in some areas. In the primary hepatocytes treated with 15 *μ*g/mL SHE78-7, cadherin adherent junctions were lost entirely in most areas (Figure 6(a)). As previously reported [24], SHE78-7 caused a concentration-related disruption in cadherin adherent junction. SHE78-7 at 15 *μ*g/mL had a better suppressive effect on cadherin adherent junctions and was used in the subsequent experiments.

Compared with the control cells stimulated by 500 × *g* and untreated with SHE78-7, the relative luciferase activities of HNF4*α* P1 in primary hepatocytes exposed to 15 *μ*g/mL SHE78-7 and stimulated by 500 × *g* were 12.2% (*P* = 0.065) lower at 3 h and 30.4% (*P* < 0.05) lower at 24 h (Figure 6(b)). The relative luciferase activities of HNF4*α* P2 were 17.4% (*P* = 0.072) lower at 3 h and 18.1% (*P* < 0.05) lower at 24 h (Figure 6(c)).

Moreover, in the primary hepatocytes treated with 15 *μ*g/mL SHE78-7 and stimulated by 500 × *g*, the protein expression level of HNF4*α* was 10.3% (*P* = 0.087) lower than that of the control cells untreated with LatB and stimulated by 500 × *g* at 3 h and 22.3% (*P* < 0.01) lower than that at 24 h (Figures 6(d) and 6(e)). The mRNA expression level of HNF4*α* was 10.8% (*P* = 0.058) lower at 3 h and 29.8% (*P* < 0.001) lower at 24 h (Figure 6(f)).

### 3.5. Disruption of F-Actin Polymerization and E-Cadherin-Mediated Cell Adherent Junctions Partially Suppresses Primary Hepatocyte Functions Stimulated by Mechanical Compaction

**Figure 7:**
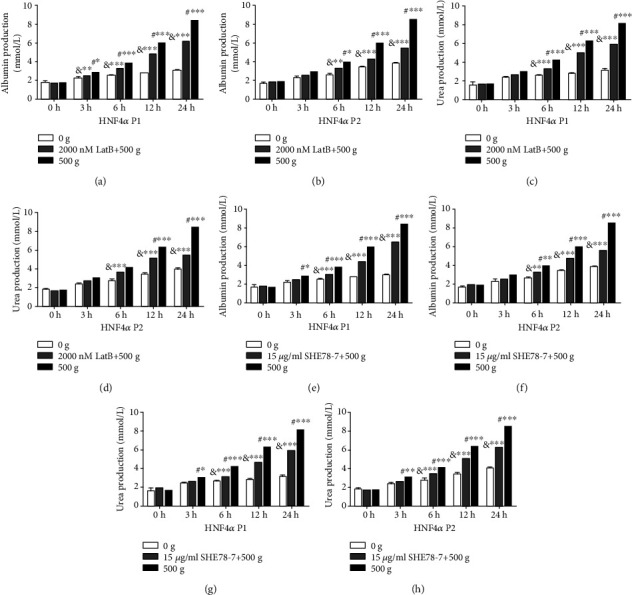
Disruption of F-actin polymerization and E-cadherin-mediated cell adherent junctions partially weakens primary hepatocyte functions triggered by mechanical compaction. (a–h) The levels of albumin and urea production in primary hepatocytes exposed to LatB or SHE78-7 and stimulated by 500 × *g* were lower than those in cells stimulated by 500 × *g* and untreated with LatB or SHE78-7 but still higher than those in cells just stimulated by 0 × *g* (two-way ANOVA analysis and Bonferroni post-tests, ^&^LatB or SHE78-7-treated vs. 0 g, ^#^LatB or SHE78-7-treated vs. 500 g, *n* = 3, ^∗^*P* < 0.05, ^∗∗^*P* < 0.01, ^∗∗∗^*P* < 0.001).

Compared with the control cells stimulated by 500 × *g* and untreated with LatB, the albumin production in primary hepatocytes transfected with clones containing the HNF4*α* P1 or P2 gene sequences, exposed to 2000 nM LatB and stimulated by 500 × *g*, was, respectively, 12.8% (*P* < 0.05) and 12.5% (*P* = 0.057) lower at 3 h and 26.2% (*P* < 0.001) and 35.7% (*P* < 0.001) lower at 24 h (Figures 7(a) and 7(b)). The urea production was 11.0% (*P* = 0.063) and 10.9% (*P* = 0.059) lower at 3 h and 26.7% (*P* < 0.001) and 35.0% (*P* < 0.001) lower at 24 h (Figures 7(c) and 7(d)).

Similarly, compared with the control cells stimulated by 500 × *g* and untreated with SHE78-7, the albumin production in primary hepatocytes transfected with clones containing the HNF4*α* P1 or P2 gene sequences, exposed to 15 *μ*g/mL SHE78-7 and stimulated by 500 × *g*, was, respectively, 12.8% (*P* < 0.05) and 13.6% (*P* = 0.056) lower at 3 h and 23.0% (*P* < 0.001) and 34.5% (*P* < 0.001) lower at 24 h (Figures 7(e) and 7(f)). The urea production was 13.2% (*P* < 0.05) and 15.2% (*P* < 0.01) lower at 3 h and 27.2% (*P* < 0.001) and 26.8% (*P* < 0.001) lower at 24 h (Figures 7(g) and 7(h)).

### 3.6. Overexpression of HNF4*α* Accelerates BC-Like Lumina Formation and Maturation in HepG2 Cells

To investigate whether the enhanced or suppressed expression of HNF4*α* can be involved in hepatic polarization, the HNF4*α* expression in HepG2 cells was manipulated by infection with Ad-HNF4*α* or LV-HNF4*α* and Ad-Mock or LV-Mock as the blank control, of which the effects were verified at both the mRNA and protein levels. The detailed results are shown in supporting Figure S2.

**Figure 8:**
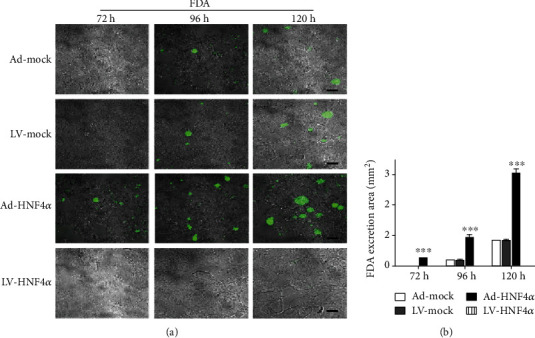
HNF4*α* accelerates the functional formation of HepG2 cell polarization. (a, b) Excretion of FDA and the areas of FDA localization increased significantly over time in Ad- or LV-HNF4*α* transfected HepG2 cells (two-way ANOVA analysis and Bonferroni posttests, Ad-HNF4*α* vs. Ad-Mock, LV-HNF4*α* vs. LV-Mock, *n* = 3, ^∗∗∗^*P* < 0.001). Ad-Mock: adenovirus control vector; LV-Mock: lentivirus control vector; Ad-HNF4*α*: the recombinant HNF4*α* gene adenovirus expression vector; LV-HNF4*α*: the oligonucleotide encoding short hairpin HNF4*α* RNA recombinant lentivirus; FDA: fluorescein diacetate. Scale bars: 20 *μ*m.

The excretion of fluorescent bile acid analogues or organic ions, such as FDA, is a common method to explore functional polarity in cultured cells [25–27]. In the HepG2 cells with HNF4*α* overexpression, FDA secretion began at 72 h postseeding and FDA filled BC-like lumina between cells (Figure 8(a)). The areas of secreted FDA were 0.25 ± 0.03 mm^2^ at 72 h postseeding and increased to 3.04 ± 0.13 mm^2^ at 120 h postseeding (*P* < 0.001, Figure 8(b)); the metabolism and excretion of FDA both increased in a time-dependent manner. In the HepG2 cells with the HNF4*α* expression suppressed, we did not observe the formation of BC-like lumina even at 120 h postseeding. In the control HepG2 cells, FDA secretion began at 96 h postseeding, and the areas of secreted FDA were lower than those in cells with HNF4*α* overexpression.

## 4. Discussion

Our data suggest that HNF4*α* can be actively involved in the hepatic polarization in the context of environmental mechanical compaction, which is associated with the actin cytoskeleton and cadherin-mediated cell adherent junctions.

In our study, centrifugation was used to accumulate cells with an increased packing density and contact before static culture. Cell seeding is an important step in tissue engineering, and various cell seeding methods have been studied nowadays [28–31]. External force seeding is one of these methods, such as centrifugation [32, 33] and filtration seeding [34]. Centrifugation seeding is demonstrated to be superior to static and spinner flask methodologies in terms of seeding efficiency and cellular distribution within scaffolds [35, 36]. Using centrifugation seeding, a high seeding efficiency of up to 90% can be achieved in a short seeding time of 3-5 min for 1000-1800 rpm. Centrifugation of cells imposes an external force, and this force within a range of <2000 rpm, which we normally perform in mammalian cell culture, does not cause damage to cells [36]. Therefore, here, we used centrifugation to perform the mechanical force on cells. Moreover, when the same treatment to primary hepatocytes is applied, we found that the relative luciferase activities of HNF4*α* P1 and P2 induced by centrifugation were similar to those of the HEK-293T cells. The results indicated that the direct activation of HNF4*α* P1 and P2 was independent from cell type, and using centrifugation to perform mechanical force on cells is an effective method.

A previous study has found that mechanical compaction can accelerate hepatocyte polarization [4]; however, the cellular mechanism underlying the effect is mostly unclear. In the present study, HNF4*α* expression was significantly elevated with mechanical compaction clearly verified by luciferase reporter assay in both nonhepatic and hepatic cell models and then by tests at the mRNA and protein levels, therefore supporting the argument that mechanical compaction can directly activate HNF4*α* expression. HNF4*α* has been showed to be crucial for hepatic polarization in liver morphogenesis [12, 13, 37]; therefore, we went on determining whether HNF4*α* is involved in the accelerated hepatic polarization. We established HepG2 cell lines with both enhanced and suppressed HNF4*α* expression and found that the enhanced expression of HNF4*α* can accelerate HepG2 cell polarization, while the suppressed HNF4*α* expression inhibited HepG2 cell polarization. These findings indicated that, although there can be several pathways underlying the accelerated hepatic polarization by mechanical compaction, it is probable that mechanical compaction directly promotes the HNF4*α* expression which then affiliates the preparation of polarization-related pathways inside the hepatocyte. Meanwhile, mechanical compaction also produced the enhanced contact between the cells, which together with the intracellular preparation accelerates the polarization formation between the hepatocytes. Nevertheless, the accelerated polarization can be physiological as we showed that the presence of the functional structure of polarity (BC) and the level of typical hepatic production function (albumin and urea) were consistent with polarity acceleration.

In addition, we also found that the cell-cell contacts stimulated by mechanical compaction had positive effects on the expression of HNF4*α*. When the F-actin polymerization or cadherin-mediated adherent junctions were disrupted, which are two essential molecules of the cell-cell contact formation, the activation of HNF4*α* by mechanical compaction was partially and significantly suppressed. This was possibly due to the interrupted transduction of mechanical stimuli to promote HNF4*α* expression or the blockage of F-actin and E-cadherin which could directly suppress the expression of HNF4*α* or its related upstream regulatory molecules. We also found that suppressed activation of HNF4*α* was associated with the weakened polarization of primary hepatocytes and their production function. Thus, all these demonstrate that the mechanical compaction, promotion of HNF4*α* expression, and accelerated hepatic polarity are indeed closely associated.

It is well known that hepatocytes are a type of specialized epithelial cell with unique hepatic polarity phenotype in vivo. Hepatic polarity is the important key factor for the generation and maintenance of physiological function in hepatocytes [2]. Much effort is taken to mimic the hepatic polarity morphology when culturing hepatocytes in vitro so as to maintain proper hepatic functions as long as possible. Specifically, for hepatocytes cultured in vitro, they entered a flattened morphology, remain nonpolarized, and rapidly lose their liver-specific functions [2]. Therefore, hepatocytes are considered as fragile cells, and it is important to explore how to tune the microenvironment properly in order to maintain or enhance hepatic differentiation and function. Under experimental conditions, hepatocytes have been found to alter their multicellular structure like BC to enhance the hepatic functions when they experience mechanical stimuli such as surface binding affinity, aggregate size, substrate rigidity, and mechanical compaction [4, 38, 39]. Moreover, it became evident that mechanical forces applied by the pericellular environment or generated inside cells function as important upstream signals that trigger the establishment of cell and tissue polarity [5–7]. In this study, centrifugation was used to perform mechanical force on hepatocytes. As a result of centrifugation-generated forces, hepatocytes undergoing compaction not only exhibited accelerated repolarization, an in vivo-like morphology, but also better maintained hepatic functions as compared to the same cells cultured without centrifugation. As a novel method to modulate cell compaction and intercellular interactions, using instant centrifugation to simulate the mechanical compaction on the cell is a promising approach to maintain cultured hepatocyte in vitro at an in vivo-like morphology without the need to apply any additional processing.

HNF4*α* is a transcription factor essential for expression of a large array of genes that define hepatocyte function; for example, genes define the synthetic, metabolic, and detoxifying functions of the hepatocyte [40]. Hepatocytes from the liver deficient in HNF4*α* were small, failed to make proper cell-cell contacts, and lacked normal BC [12]. In the study, we found that mechanical compaction directly promotes the HNF4*α* expression to accelerate hepatocyte polarization. Since HNF4*α* is a key important regulator of the physiological function of hepatocytes, this further illustrates that mechanical compaction is a promising approach to maintain the function of cultured hepatocytes in vitro.

As the main cell type in the liver, hepatocytes are responsible for detoxification, metabolism, bile and protein production, and so on [41]. In vitro cultured hepatocytes are widely used in regenerative medicine, tissue engineering, drug testing platforms, and biological mechanism studies. Now, we have established a model to maintain the function of cultured hepatocytes in vitro; in the future studies, we can explore the longest time of the model for maintaining hepatocyte function and the possibility of using the model for drug toxicology testing.

## 5. In Conclusion

Our data demonstrate the first direct evidence that mechanical compaction can accelerate hepatocyte polarization through activating HNF4*α*, and the HNF4*α* activation can be associated with cell-cell contacts modulated by the actin cytoskeleton and cadherin-mediated cell adherent junctions. Our study provides an interesting insight into the molecular pathway underlying the physiological hepatocyte polarization associated with the extracellular environmental mechanical force. Future investigation is needed to identify the other important pathways in addition to the HNF4*α* activation for maintaining the normal hepatic polarization by environmental mechanical force, which can be meaningful for the biological understanding as well as the liver medicine. In addition, we have established a model to maintain the function of cultured hepatocytes in vitro. In the future studies, we can explore the longest time of the model for maintaining hepatocyte function and the possibility of using the model for drug toxicology testing, tissue engineering, biological mechanism studies, and so on.

## Data Availability

The data used to support the findings of this study are available from the corresponding author upon request.

## Supplementary Materials

Supplementary Materials Figure S1: mechanical compaction has no damage on the cell viability. Figure S2: HNF4*α* expression of HepG2 cells could be effectively modulated by Ad- or LV-HNF4*α* recombination vector.
