# Supplementary material for: Hepatic Polarization Accelerated by Mechanical Compaction Involves HNF4α Activation
Source: Biomed Res Int. 2020 Aug 5;2020:8016306. doi: 10.1155/2020/8016306 (PMC7426769; doi:10.1155/2020/8016306)
Supplement: Supplementary Materials — Figure S1: mechanical compaction has no damage on the cell viability. Figure S2: HNF4α expression of HepG2 cells could be effectively modulated by Ad- or LV-HNF4α recombination vector. [file 8016306.f1.docx]

**Hepatic polarization accelerated by mechanical compaction involves HNF4α activation**

Jinlian Yang^2,3^, Jiaen Liang^2,3^, Yongjian Zheng^2,4^, Shiying Li^2,3^, Yang Li^4^, Haiyan Liu^4^, Guanzhong Chen^4^, Jing Ma^4^, Ziyu Liao^4^, Jiezhao Lin^4^, Zesheng Jiang^4^ and Yan Wang^1,2,3*^

Jinlian Yang: yjl1190@163.com; Jiaen Liang: 595562632@qq.com; Yongjian Zheng: 13265003359@163.com; Shiying Li: 1170930700@qq.com; Yang Li: liyang2343@163.com; Haiyan Liu: 183053307@qq.com; Guanzhong Chen: 295145414@qq.com; Jing Ma: 464857575@qq.com; Ziyu Liao: 1019280693@qq.com; Jiezhao Lin: 308873816@qq.com; Zesheng Jiang: jzsdxx@163.com; * Yan Wang: yanwang@smu.edu.cn

^1^ State Key Laboratory of Organ Failure Research, Guangdong Provincial Research Center for Liver Fibrosis, Department of Infectious Diseases and Hepatology Unit, Nanfang Hospital, Southern Medical University, Guangzhou, Guangdong 510515, China;

^2^ Biomedical Research Center, Southern Medical University, Guangzhou, Guangdong 510515, China;

^3^ School of Pharmaceutical Sciences, Southern Medical University, Guangzhou, Guangdong 510515, China;

^4^ Department of Hepatobiliary Surgery, Zhujiang Hospital, Southern Medical University, Guangzhou, Guangdong 510280, China.

****Correspondence:***

Yan Wang MD, PhD

State Key Laboratory of Organ Failure Research, Guangdong Provincial Research Center for Liver Fibrosis, Department of Infectious Diseases and Hepatology Unit, Nanfang Hospital; Biomedical Research Center, Southern Medical University

**Address:** No. 1023, South Shatai Road, Baiyun District Guangzhou, Guangdong 510515, China

**Supplementary Materials**

**
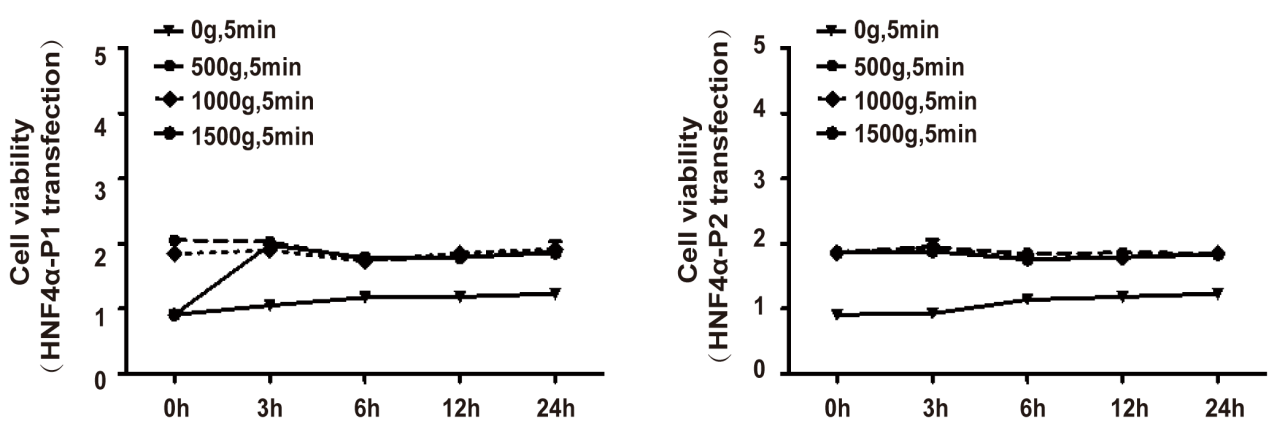
**

**Figure S1: Mechanical compaction has no damage on the cell viability.** Cell viabilities and HNF4α- P1 and P2 relative luciferase activities of HEK-293T transfected with recombination vectors of HNF4α- P1 and P2 promoter and dual luciferase genes and treated with 500-1500g force. All data were expressed as mean ± standard deviation (mean ± S.D.).

**
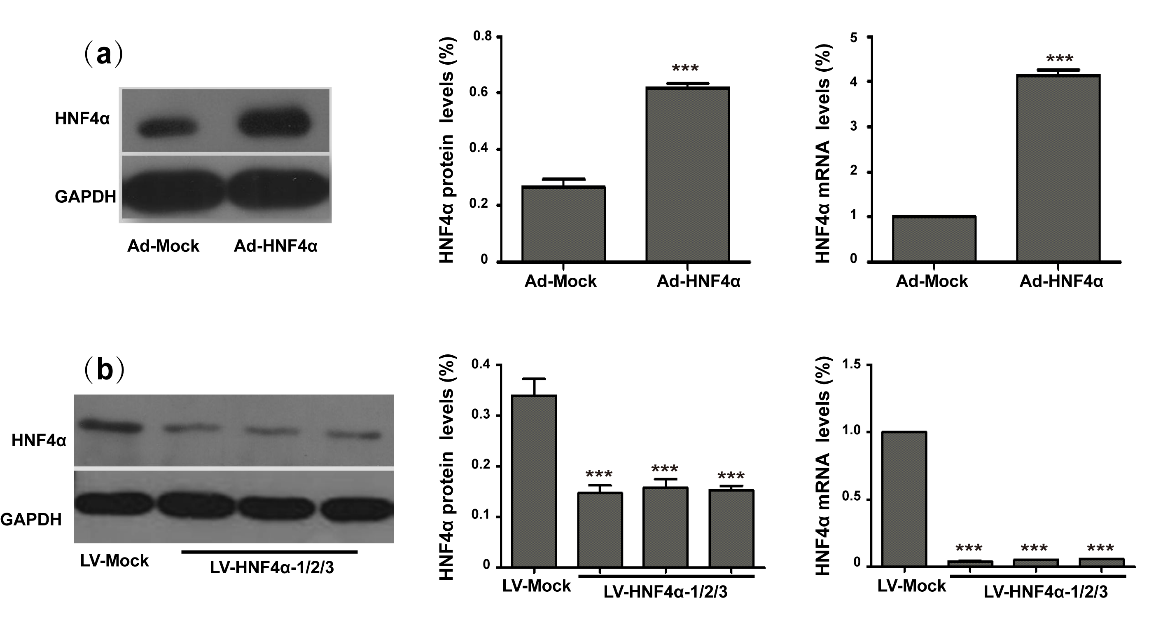
**

**Figure S2: HNF4α expression of HepG2 cells could be effectively modulated by Ad- or LV-HNF4α recombination vector.** (a) The mRNA and the protein expression levels of HNF4α of HepG2 cells transfected by Ad-HNF4α (Student’s t-test, Ad-HNF4α vs. Ad-Mock, n=3, ****P*<0.001). (b) The mRNA and the protein expression levels of HNF4α of HepG2 cells transfected by LV-HNF4α (one-way ANOVA analysis and Dunnett's Multiple Comparison Test, LV-HNF4α vs. LV-Mock, n=3, ****P*<0.001).
